# Pure Curcumin Spherulites from Impure Solutions *via* Nonclassical Crystallization

**DOI:** 10.1021/acsomega.1c02794

**Published:** 2021-09-02

**Authors:** K. Vasanth Kumar, Kiran A. Ramisetty, K. Renuka Devi, Gamidi Rama Krishna, Claire Heffernan, Andrew A. Stewart, Jian Guo, Srinivas Gadipelli, Dan J. L. Brett, Evangelos P. Favvas, Åke C. Rasmuson

**Affiliations:** † Synthesis and Solid State Pharmaceutical Centre, Department of Chemical Sciences and the Bernal Institute, University of Limerick^RINGGOLD^, Limerick V94 T9PX, Ireland; ‡ Department of Physics and the Bernal Institute, University of Limerick, Limerick V94 T9PX, Ireland; § Department of Chemistry, University College London, 20 Gordon Street, London WC1H 0AJ, U.K.; ∥ Electrochemical Innovation Lab, Department of Chemical Engineering, University College London, Torrington Place, London WC1E 7JE, U.K.; ⊥ Institute of Nanoscience and Nanotechnology, NCSR “Demokritos”, Agia Paraskevi 15341, Attica, Greece

## Abstract

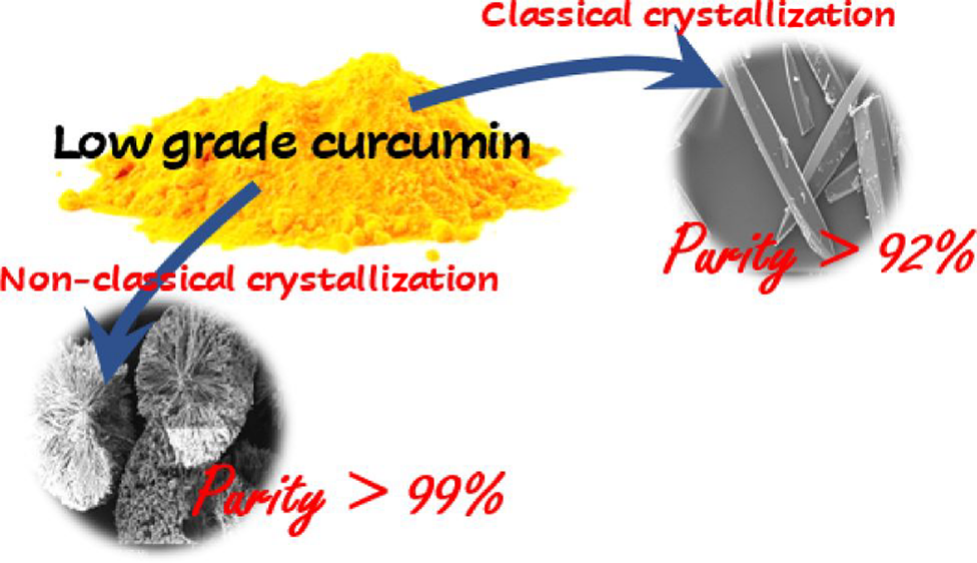

Crystallization experiments performed with highly supercooled solutions produced highly pure (>99 wt %) and highly crystalline mesocrystals of curcumin from impure solutions (∼22% of two structurally similar impurities) in one step. These mesocrystals exhibited a crystallographic hierarchy and were composed of perfectly or imperfectly aligned nanometer-thick crystallites. X-ray diffraction and spectroscopic analysis confirmed that the spherulites are a new solid form of curcumin. A theoretical hypothesis based on particle aggregation, double nucleation, and repeated secondary nucleation is proposed to explain the spherulite formation mechanism. The experimental results provide, for the first time, evidence for an organic molecule to naturally form spherulites without the presence of any stabilizing agents. Control experiments performed with highly supercooled pure solutions produced spherulites, confirming that the formation of spherulites is attributed to the high degree of supercooling and not due to the presence of impurities. Likewise, control experiments performed with a lower degree of supercooling produced impure crystals of curcumin *via* classical molecular addition mechanisms. Collectively, these experimental observations provide, for the first time, evidence for particle-mediated crystallization as an alternate and efficient method to purify organic compounds.

## 1 Introduction

Crystallization is one of the most important techniques for the separation and purification of molecules of industrial importance. Crystallization is an inherently multiscale process, in which subtle changes in how molecules interact can give rise to distinct structures on the mesoscale and consequently macroscopic (bulk) properties of the material.^1−3^ As opposed to the conventional way of monomer-by-monomer addition, crystallization can proceed *via* particle-based reaction channels.^3−6^ Particle-mediated crystallization, also called as nonclassical crystallization, refers to the mesoscopic transformation of self-assembled, metastable or amorphous precursor particles into three-dimensional superstructures or mesocrystals with complex morphologies.^1,7,8^ Exposing the mechanisms of the formation of mesocrystals is challenged by the lack of suitable techniques that can capture the crystallographic fingerprint of the subunits over different length and time scales. Recently, the term mesocrystal has been redefined to describe these superstructures based on their unique morphological features rather than on the formation mechanism.^9^ Mesocrystals refers to a superstructure of crystalline nanoparticles with external faces on the scale of a few hundred nanometers to micrometers.^8^ Since the term mesocrystal was coined, only a few hundred mesocrystals have been identified with their formation mechanisms hypothesized.^9^ Earlier, mesocrystal-like structures were usually classified as anisotropic polycrystalline materials such as fibers, polymeric films, and rolled metals.^10^

Although mesocrystals are a fascinating class of crystalline materials, practical applications of these structures are limited, and their formation mechanisms or the ways to control their final properties, such as size and shape, are not well understood. Identification of any new molecules that can evolve into mesocrystals is still welcomed, as they can serve as a new system to extract information on the structural evolution of mesocrystals over different length or time scales. Additionally, they can help to bring new evidence to trace the mechanistic pathways involved in the much-disputed topic of nonclassical crystallization processes. It can also help to bring new explanations for some of the similar processes that occur naturally (*e.g.*, biomineralization), leading to advanced artificial methods.^11^ Mesocrystals are most frequently encountered in systems where high cohesion energies are involved, especially in mineralization from viscous magmas, inorganic compounds from melts that contain thickeners, inorganic crystals crystallized from impure solutions, and substances that seem to exist as liquid crystals at some specific temperatures.^12^ More recently, Wohlrab reported the alignment of the first organic molecule dl-alanine crystals in the presence of a polymer, and mesocrystals were assumed to assemble *via* the oriented assembly of polymer-stabilized nanocrystals.^13,14^

In this work, we have found the formation of organic mesocrystals of an industrially important and neutral molecule, curcumin, in isopropanol (IPA) from its supercooled solution that contains >20 wt % of two structurally similar impurities, bisdemethoxycurcumin (BDMC) and demethoxycurcumin (DMC), without the presence of any stabilizing agents in the solution. Additionally, these mesocrystals are pure (>99%) with remarkably narrow particle size distributions. This is a striking result, considering that our earlier work showed that at least four consecutive recrystallization steps are required to achieve this level of purity.^14^ Purification of organic compounds in the presence of structurally similar impurities is always considered to be a big challenge, as here, the impurities can incorporate *via* at least two different mechanisms—(i) lattice replacement and (ii) equilibrium or non-equilibrium adsorption of impurities; both mechanisms occur during the growth *via* the classical pathway “*molecule-by-molecule*” addition and particle coarsening. In our earlier work, as mentioned above, curcumin crystallized from impure solutions following these classical pathways but produced impure crystals or required multiple recrystallization steps to obtain a pure product.^15^ Alternatively, as shown here, if we force the crystallization to proceed *via* particle aggregation, it is possible to produce a pure product in a single step.

The central purpose of this article is to show that particle aggregation-driven crystallization can serve as an alternate and novel approach to produce pure solid products of curcumin from its lower grades. The scientific approach here is to find the conditions under which the crystallization can be forced into a particle aggregation mechanism without the inclusion of impurities, rather than *via* particle coarsening and poor impurity separation. Here, the term “particle aggregation” refers to particle formation by perfectly or imperfectly associating crystallites into larger solid aggregates. Another main objective of this work is to perform critical analysis through characterization of the bulk properties of curcumin spherulites using chromatographic [high-performance liquid chromatography (HPLC)], thermal [differential scanning calorimetry (DSC)], microscopic [scanning electron microscopy (SEM), transmission electron microscopy (TEM), and light microscopy], diffraction [powder X-ray diffraction (PXRD) and single-crystal X-ray diffraction (SCXRD) and small-angle X-ray scattering (SAXS)], and spectroscopic (Raman) techniques. To date, most of the studies in the mesocrystal literature, especially the ones dealing with organic molecules, limit their focus to solid-state characterization of the final crystals to hypothesize the formation mechanisms.^8^ Additionally, most of these studies identified the formation mechanism of mesocrystals from pure solution or melts or in the presence of a purposely added stabilizing agent. Only one system has been identified so far where particles of organic molecules grow *via* particle attachment without any stabilizing agent, although the final product is not completely crystalline.^16^ To the best of our knowledge, the present study is the first experimental work that shows lower grade curcumin precipitating into pure and highly crystalline mesocrystals *via* nonclassical routes or into impure but stable single crystals depending on the initial conditions and more specifically without the presence of any stabilizing agents. This first-time experimental observation allowed us to propose several new theoretical hypotheses for the formation of stable and pure mesocrystals from impure solutions. Additionally, we also expose the existence of several complex structures of the intermediate phase that precedes the formation of mesocrystals using microscopic analysis. A hypothesis is proposed to explain why the low-grade curcumin precipitates into pure mesocrystals. We also identify that mesocrystals have the property to self-regulate the particle size, which has not been reported in the mesocrystal literature. The results presented here and the mechanisms identified/proposed can serve as a useful and new strategy to purify organic compounds *via* nonclassical crystallization. The results will also expose curcumin as a versatile system that can produce either mesocrystals or single crystals from their solutions depending on the initial conditions, without the use of chemical agents. In addition, a theoretical hypothesis is proposed to explain why the low-grade curcumin precipitates into pure mesocrystals.

## 2 Results and Analysis

### 2.1 Crystallization Kinetics of Curcumin from Pure and Impure Supercooled Solutions, Product Purity, and Particle Morphology

**Figure 1:**
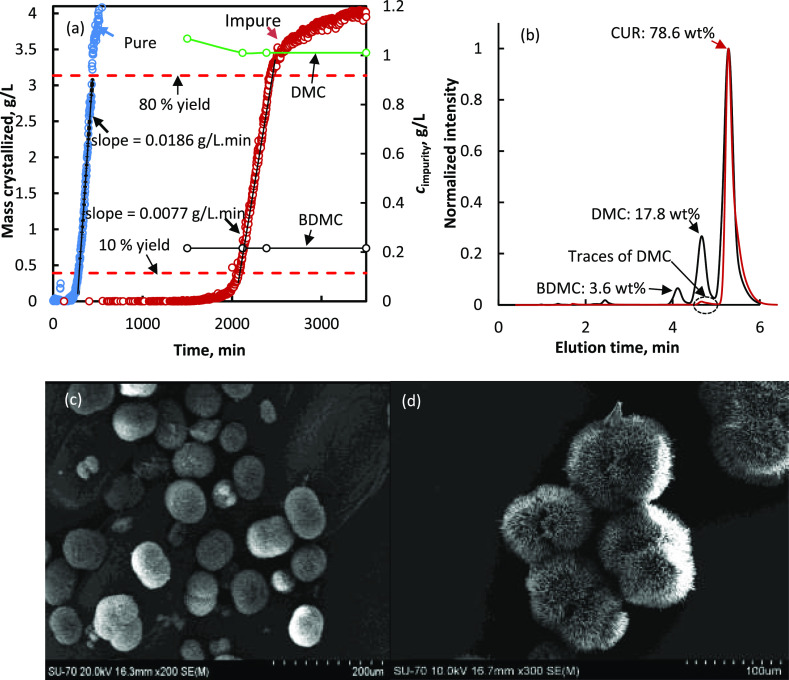
(a) Crystallization kinetics of curcumin at 5 °C from a highly supercooled (Δ*T* = T* – *T*_w_ = 55 °C) impure (that contains 1.068 g L^–1^ DMC and 0.216 g L^–1^ BDMC; open red circles) and pure solution (blue open circles); also shown is the depletion in the concentration of impurities DMC and BDMC obtained by HPLC during crystallization; (b) HPLC chromatogram of the final products obtained from highly supercooled impure solutions (red lines); purity of the crude product used to prepare the impure solution is also shown for comparison (black lines); and (c) SEM image of the final products obtained from highly supercooled impure solution (Δ*T* = 55 °C) and (d) from highly supercooled pure solution (Δ*T* = 55 °C).

Figure 1a shows the plot of mass crystallized *versus* time during the crystallization of curcumin spherulites from a highly supercooled (Δ*T* = 55 °C) pure and crude curcumin solution at 5 °C. It can be seen that the crystallization onset time was significantly altered by the presence of impurities. The onset of the crystallization is approximately eight times faster during the formation of spherulites from the pure solution. Notably, in both cases, the solution has reached the final temperature of 5 °C before the onset of crystallization can be observed. The delay in the onset of crystallization in impure solutions can be associated with the tendency of the impurity molecules to interfere and break the self-assembling pattern of the pre-nucleation clusters to form the stable nuclei and thus the primary crystallite.^17^Figure 1b shows the HPLC chromatogram of the final product (Figure 1b) obtained from an impure curcumin solution (black) and compares to that of the crude material (red). The crystallized product obtained from the impure solution is pure (>99% purity). DMC appears in trace quantities, but there is no BDMC in the final solid product. The high purity of the material corresponds to the monitoring of the solution impurity concentrations (see Figure 1a), during crystallization. There is a very small decay in the DMC concentration and no detectable change in the BDMC concentration. Figure 1c,d shows SEM images of the final product. Regardless of whether the solution is pure or impure, under the conditions used, that is, a high initial supersaturation and a crystallization temperature of 5 °C, the product has spherical particle morphology.

**Figure 2:**
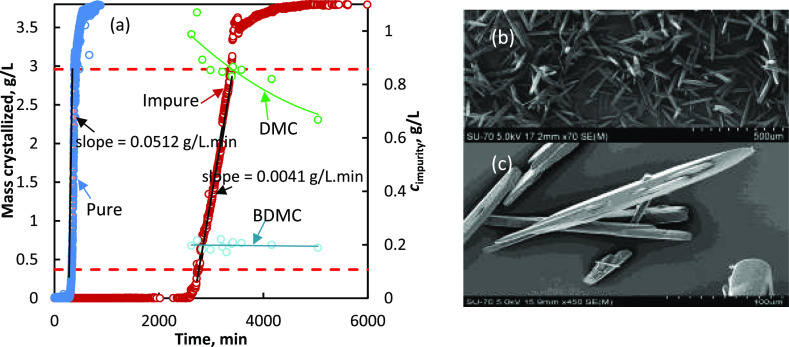
(a) Crystallization kinetics of curcumin at 25 °C from a less-supercooled (Δ*T* = 35 °C) pure solution and impure solution that contains 1.068 g L^–1^ DMC and 0.216 g L^–1^ BDMC; also shown are the depletion in the concentration of the impurities, DMC and BDMC, in the solution and SEM image of the final crystals obtained from low-supercooled (Δ*T* = 35 °C) (b) impure solution and (c) pure solution. Note: the SEM images in Figures 1 and 2 are captured at different magnification levels, and these images clearly show that these samples essentially contain either spherulites or needles depending on the degree of supercooling (under the studied experimental conditions, the final product contains either needles or spherulites and never a mixture of both).

In Figure 2a, the corresponding data are shown for the experiments performed with less-supercooled solutions (and thus a lower initial supersaturation; see Figure 2 for details) at 25 °C. Figure 2a shows the results expressed in terms of mass crystallized *versus* time. Also shown is the depletion in impurity concentration in the solution as a function of time. Typically, crystallization under these conditions always produced individual needle crystals of curcumin, as shown in Figure 2b,c. It can be realized from Figure 2a that the impurity concentration of the solution decreases progressively with time (Figure 2a). In terms of the final product purity, the depletion in the concentration of the impurities in the solution shows that at least one of the impurities, DMC, is continuously integrated into the crystalline material throughout the crystallization. Practically, there is no integration of the other impurity, BDMC. Up to 0.4 g of DMC is transferred into the crystallizing compound per liter of the solvent. If this value is compared with the theoretical mass of curcumin that can be crystallized (3.82 g L^–1^) under the studied experimental conditions [Δ*T* = 35 °C; *T*_w_ = 25 °C; c* at 25 °C = 0.98 g L^–1^ IPA, and c* at 60 °C = 4.8 g L^–1^ IPA], the purity of the final product should be 92% by weight. In Figure 2a, the crystallization kinetics, expressed in terms of mass crystallized *versus* time, are shown. Similar to the experiments performed with the highly supercooled solution, the onset of nucleation in pure solution was roughly 8.5 times faster than for the impure solution. The onset of nucleation in the less-supercooled solution at 25 °C is slightly longer than that in a more supercooled solution at 5 °C.

The continuous transfer of impurities into the solid phase observed during the crystallization in the less-supercooled solution is an expected phenomenon and can be explained based on adsorption theories and diffusion concepts. The product purity obtained during the cooling crystallization experiments is usually correlated to the solubility of the pure compounds and the concentration of impurities in solution at any instant of time. In impure solutions, impurities are prone to adsorb onto the crystal surfaces and compete with the target molecule during crystallization.^19,20^ The probability of solute molecule (in this case, impurity) transfer to the solid phase is high when the ratio of the concentration of impurity to the target molecule in the bulk solution is higher. On the other hand, in a highly supersaturated or supercooled solution, when the initial concentrations of the crystallizing compound are higher than the impurity concentration, it is least likely for an impurity molecule to reside on the crystal surface or to desorb the crystallizing molecule from the crystal surface. Alternatively, if the impurities are structurally similar (as in the present case) to the target compound, impurity molecules can replace the crystal lattice without significantly altering the crystal habit, irrespective of the concentration of the impurity or the target compound.^20^ This explains the depletion in the concentration of DMC in the solution during crystallization from a less-supercooled solution. During crystallization, especially at lower supersaturation, the ratio of the impurity to the leftover curcumin concentration in solution will be higher when compared to that under the initial experimental conditions. Then, the impurity molecule can compete with the target molecule for the active sites on the crystal surface, thereby altering the purity of the final product. The continuous transfer of one of the impurities, DMC (see Figure 2a), to the solid phase indicates that all of these effects could have played their role during the crystallization of curcumin single crystals from the less-supercooled solution.

The non-transfer of the other impurity, BDMC, can be attributed to the structure of this molecule. Structurally, BDMC is more different from curcumin than DMC. BDMC does not contain any −OCH_3_ group in the structure, whereas DMC contains at least one −OCH_3_ group, partly resembling the structure of curcumin (see Table 1). In this spirit, it is more probable that the DMC molecule can find space within the crystal lattice during crystallization events. Additionally, the concentration of BDMC is relatively low when compared to that of curcumin in the solution (∼17 wt % of DMC *vs* 3 wt % of BDMC). The driving force involved should not be sufficient to drive BDMC from the solution to the crystal lattice. This means that the competing bulk diffusion effects required to promote impurity incorporation *via* the crystal lattice will be comparatively lower when compared to those of DMC.

**Table 1:**
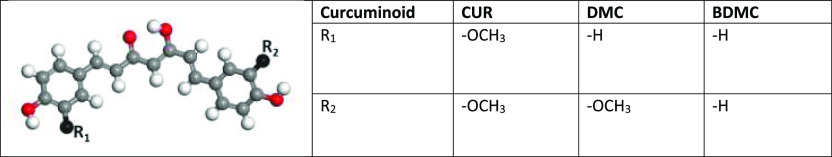
Structures of the Structurally Similar Curcuminoids CUR, DMC, and BDMC (the Groups in R_1_ and R_2_ Show the Structural Difference between the Three Curcuminoids; Atom Color, C: Gray, H: White, and O: Red)

During crystallization from a highly supercooled solution (Δ*T* = 55 °C and *T*_w_ = 5 °C), experimental results clearly showed that none of the abovementioned effects that rely on adsorption theories and molecular diffusion seem to apply during the formation of mesocrystals. The experimental conditions in Figures 1a and 2a are purposely chosen, as the earlier ones produced spherulites and the latter ones produced single crystals. In terms of mass crystallized, the amount that can be crystallized from the highly supercooled solution (Δ*T* = 55 °C) and the less-supercooled (Δ*T* = 35 °C) solution differs only by 0.22 g L^–1^ (the solubility of CUR in IPA at 5 and 25 °C is 0.76 and 0.98 g L^–1^, respectively). This also means that the ratio of curcumin that can be crystallized to the impurity concentrations in both of these experiments does not differ by a big magnitude. In this spirit,^21^ crystallization from both highly supercooled solution and less-supercooled solution should usually produce products of similar purity. In fact, the material crystallized at lower temperature and higher supersaturation would be expected to be more impure. The present results show the opposite. This is a noteworthy result, as it shows the existence of a pseudo-effect where impurity incorporation is dictated by the properties of the solids formed in the solution or possibly due to the mechanistic way these solids are formed in the solution rather than the solution properties, including the initial concentration of impurities and the temperature. Rather, we propose that the purity depends on the surface properties of the solids formed and the particle formation kinetics and mechanisms involved.

The difference in the purity level of the spherulites and the needle-shaped crystals and their overall morphology itself can also be explained based on the mechanisms that drive the crystallization process. In terms of the product morphology, it is clear from the SEM images in Figures 1 and 2 that the working temperature and the degree of supercooling dictate the final structure of the crystallized materials. The highly supercooled solution produced spherulites which are pure, and the less-supercooled solution produced crystals with an equilibrium habit but a significant number of impurities. From a kinetic standpoint, this particular temperature- and degree of supercooling-dependent effect can be taken as an analogue of the chemically and entropy-controlled self-assembling process.^21^ During cooling or isothermal crystallization, if the process is chemically controlled, the molecules tend to adsorb in a specific pattern (driven by the force fields involved) on the surface followed by its integration into the crystal lattice. Typically, a chemically controlled process should lead to crystals with an equilibrium habit.^21^ Alternatively, the temperature, degree of supercooling, or any other factors, including a high level of supersaturation, can disturb these mechanistic events, and in that case, the process will be entropically controlled. If entropic control is weak, the crystallization can be considered to occur *via* ion-by-ion or molecule-by-molecule addition through van der Waals and long-range interactions. If the process is dominated by entropic control, it is more likely that the molecule-by-molecule attachment process is disturbed, and the solute molecule will assemble *via* a nonclassical mechanism. In both cases, the impurity transfer mechanism will also be altered during the crystallization process. Clearly, the crystallization from a highly supercooled solution should have followed a different crystallization pathway to the one facilitating crystallization from a lower supercooled solution, which makes these materials pure (see Section 3, where we propose a possible mechanism based on the concepts of double nucleation/repeated 2D nucleation for the formation of pure spherulites from impure solutions). As the spherulites obtained from a highly supercooled solution do not represent the equilibrium habit of the curcumin, we presume that the crystallization in the highly supercooled solution is entropically driven, which also leads to more pure solid materials. It is often assumed that spherulites are formed *via* an unconventional growth mechanism due to the presence of impurities. However, if we compare the SEM images in Figures 1 and 2, both pure and impure solutions can crystallize into single crystals or as spherulites depending on the experimental conditions. In a later section, we explained the formation of spherulites based on the intrinsic properties of the supercooled solution. It is essential to highlight here that crystal shape and size control are usually considered to be premium crystal attributes in industrial crystallization processes. Our results show that crystallization under extreme cooling conditions forms spherulite-shaped crystals that are pure (>99% purity). Based on our results, it is possible to conclude that pure curcumin can be produced in a single crystallization step. In the present case, crystallizing from a supercooled solution appears to skip the crystal growth by the regular molecule-by-molecule attachment process and can produce pure crystals in a single step without using any toxic additives. Additionally, all the spherulites naturally grow to a specific size with a narrow size distribution in the recorded SEM images (see Figure 1c,d). This means that spherulites have the tendency to self-regulate size.

### 2.2 Detailed Product Morphology Analysis

**Figure 3:**
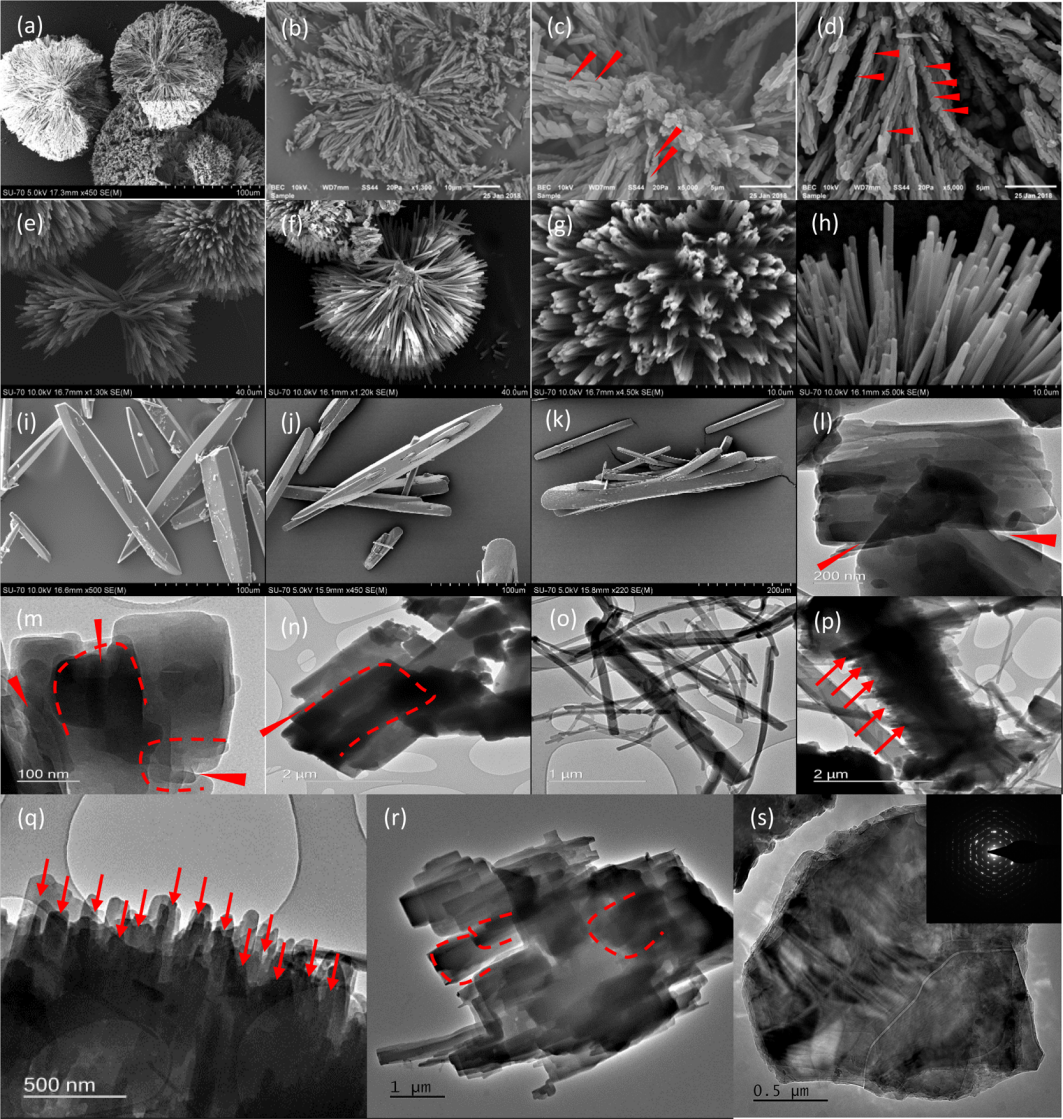
(a–d) SEM images of the spherulites obtained from a highly supercooled impure solutions; the magnified images of the spherulite core and the filament show several crystallite dockings and solid interfaces (for convenience of the readers, we show a few of the solid–solid interfaces using red daggers); (e–h) SEM images of the spherulites obtained from pure solutions; the magnified images (g,h) show that the filaments are flawless at the micron scale; and (i) SEM image of the needle-shaped curcumin crystals obtained from the less-supercooled impure solution, (j–k) SEM images of the needle-shaped curcumin crystals obtained from the less-supercooled pure solution, (l–n) TEM images of the crystallite fragments of the spherulites obtained from highly supercooled impure solution, (o) TEM image of the crystallite fragments (essentially should be crystalline fragments) from the spherulites obtained from the pure solution, (p–s) TEM images of the crystallite fragments from the spherulites obtained from the pure solution (these are presumed to be the spherulite core based on the numerous crystallite interference pattern and closely aligned nm-thick crystallites, as such features are not visible in spherulite filaments). Note: red daggers show the crystallite docking points, and red arrows and areas bounded by dotted redlines show the interference patterns due to the coalesced growth of crystallites.

Figure 3a–h,l–s shows the SEM and TEM images of the final product material obtained from impure and pure highly supercooled solutions. For comparison, we also show the SEM images of the needle-shaped crystals obtained from the less-supercooled solution (Figure 3i–k). Clearly, the spherulites obtained from both pure and impure solutions are composed of small crystallites of different sizes (see Figure 3b–h,l–o). As a general observation, there is a spherulite core from which near-micron-sized filaments stretch radially out. The marked differences between the products obtained from pure and impure solutions are that the latter contains from several nanometers (∼200 nm) to near-micron-thick crystallites and the existence of solid–solid contacts in the presence of numerous crystallite docking points (see Figure 3b–d). Another notable and common morphological feature observed is the low-angle branching of the filaments that emerge out from the spherulite core; this low-angle branching separates each fiber from one another (Figure 3d–f). Each of the fibers stretches from the core and are separated by an angle of roughly θ = 2–3°. This specific feature was observed in the spherulites formed from both pure and impure solutions. One possible explanation for the observation of low-angle branching is the concentration of stereoirregular crystallites between the crystalline fibers, which allow them to separate by a certain distance without altering the growth rate of the fibers. Morphological defects due to the incorporation of stereoirregular crystallites or co-units were reported in the early 1960s to describe the growth of semicrystalline polymers.^22^ Typically, this stereoirregular unit corresponds to impurities.^22^ As spherulites from both pure and impure solutions exhibit this particular property, the separation between the filaments can be merely attributed to the presence of stereoirregular co-units (crystallites) or differently conformed curcumin molecules. Alternatively, this can be due to the natural preference of crystallites that emerge from the primary crystallite located at the center of the spherulite to partly retain the crystal equilibrium habit and grow as filaments.

The SEM/TEM images of the spherulites obtained from the impure solution also show that the filaments and core contain nanometer–near-micron-sized crystallites assembled or docked either perfectly (see Figure 3d) or imperfectly (see Figure 3c showing the spherulite center). At the core, the crystallites are arranged randomly, involving more than two crystallites with multiple crystallite docking points (this is visible in the TEM images). This particular behavior was earlier observed during the growth of titania particles,^6^ whereas in the filaments, docking points mostly confirm the oriented attachment of two individual crystallites (this is visible in Figure 3d), where a specific facet of one crystallite docks with the next and merges in a way extending from the core of the spherulite. This type of crystallite agglomeration and existence of a solid–solid interface contact are common phenomena in nonclassical crystallization, which usually evolve into mesocrystals rather than single crystals.^1,8^ Mesocrystals are characterized by the presence of crystallographic hierarchy and a lack of equilibrium crystal habit, yet they are crystalline.^7^ The microscopic images show that curcumin spherulites have this particular mesocrystal property and crystallographic hierarchy. The spherulites are roughly 80 μm, composed of several crystallites that range from a few nm to near-microns, and the filaments are all of a similar length (20–30 μm). In terms of the structure, they do not exhibit any equilibrium crystal habit. The combination of morphological properties indicates that such structures might have formed *via* a nonclassical crystallization pathway. TEM/SEM images of spherulites obtained from the impure solution show additional features such as the crystallite interference patterns at the nanoscale due to the way the crystallites are docked toward their growing face and toward the *c*-axis (see Figure 3i–n). TEM images of the filaments (Figure 3m,n) further confirmed the coalesced growth of two crystallites. The interference pattern confirms crystallite thickening *via* stacking of crystallites toward the *c*-axis.

In the case of spherulites obtained from the pure solution, few of the above-said observations can be visualized in SEM/TEM images at the studied resolution. The filaments in the spherulites obtained from the pure solution mostly contain flawless and smooth surfaces at the micron scale (see Figure 3g,h,o). SEM/TEM images also show no evidence for crystallite docking in the filaments. We attribute these morphological differences between the spherulites obtained from pure and impure solutions to the impurity effect. The impurity effect on the crystal habit is a common phenomenon and is usually encountered during the crystallization of homogenous (single) crystals. This phenomenon seems to be applicable now during the crystallization of more heterogeneous structures like spherulites (see below). At the core (see Figure 3p–s), TEM images clearly showed several nanometer-thick crystallites (∼150 nm) arranged in parallel and toward the *c*-axis (Figure 3r shows crystallite docking toward the *c*-axis, thus exhibiting interference patterns), making an amorphous structure-like appearance (especially in Figure 3q). Figure 3s shows the selected area electron diffraction (SAED) pattern of the spherulite core, which confirms that the core is crystalline. It should be mentioned that the structures in Figure 3p–s are assigned as the spherulite core based on visual interpretation. The spherulite filaments observed in SEM are flawless without any solid–solid interface. If this is the case, the multiple solid–solid interfaces and the remarkably smaller size of the crystallites (see Figure 3q) involved in the docking process should represent a structure that should be indifferent to that of the spherulite filaments, which should be the core of the spherulite.

As far as the crystals obtained from the less-supercooled solutions are concerned, the curcumin needles obtained from both pure and impure solutions exhibit a similar morphology (see Figure 3i–k). Despite the needle-shaped crystals obtained from the impure solution containing a significant amount of impurities, they seem to be flawless; no solid–solid interface is observed in the case of spherulites obtained from the impure solution. This clearly shows that the solid formation mechanism should have followed a different route during the formation of spherulites and needles from highly supercooled and less-supercooled solutions. Practically, there is no evidence for the presence of solid–solid interfaces other than at the locations where we observed twinned crystals that emerge at a low angle from the middle of the needles (see Figure 3i–k). There are no traces in the SEM images to expose the formation of needles *via* particle attachment such as multiple docking points and crystallite docking toward the growing face.

Based on the abovementioned observations, the formation of spherulites can be attributed to the effect of supercooling, generating a higher supersaturation ratio (*S* = 6.2) and a lower working temperature (*T*_w_ = 5 °C).

### 2.3 PXRD, Raman, DSC, and SAXS Analysis of Spherulites Obtained from Impure Solutions

**Figure 4:**
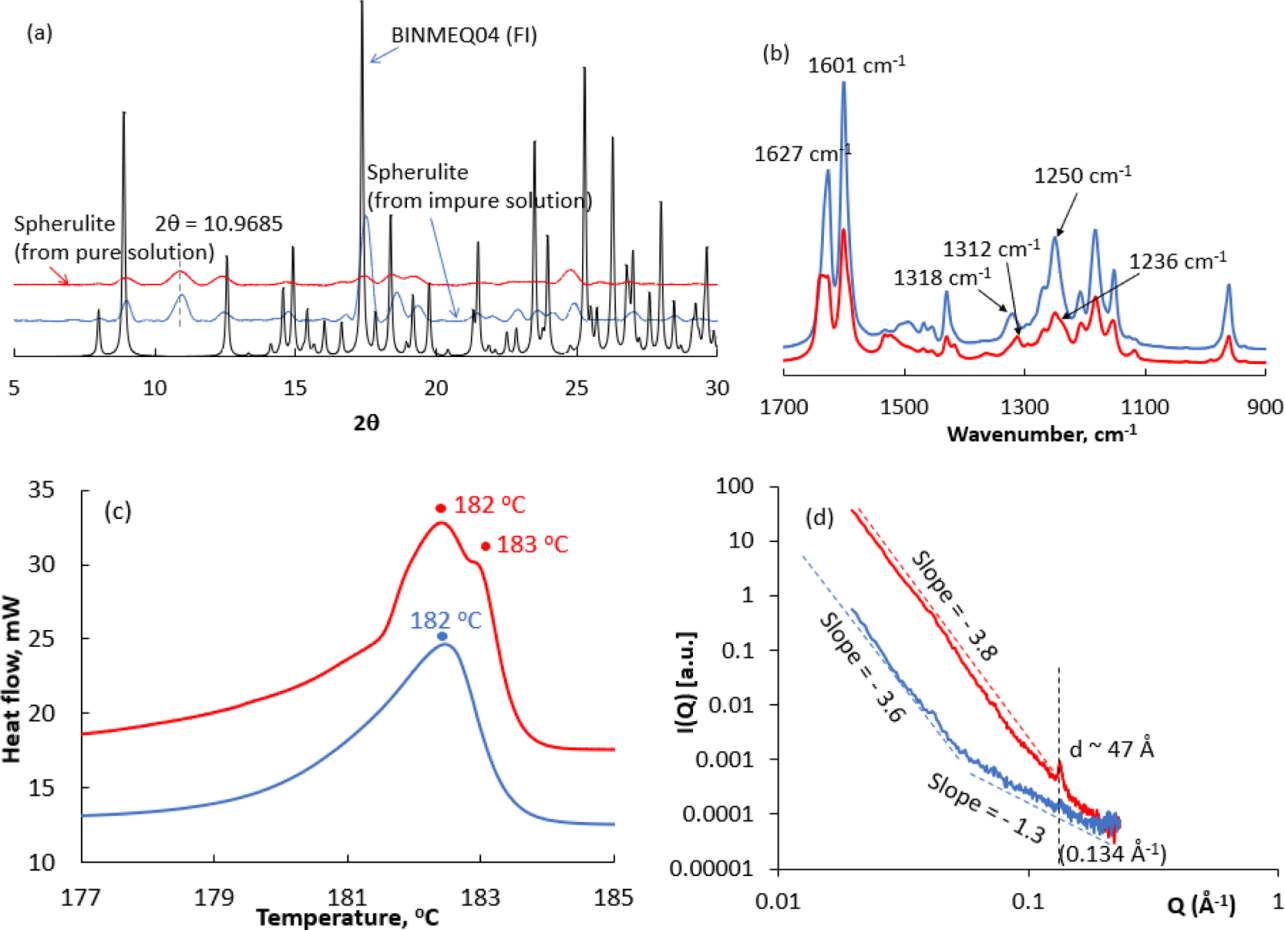
(a) PXRD of the spherulites obtained from the impure solution (blue lines) and pure solution (red lines); the simulated PXRD pattern of FI curcumin is shown for comparison (black lines); (b) Raman spectra of spherulites obtained from the impure solution (red lines) and standard FI curcumin (blue lines), (c) DSC chromatogram of spherulites obtained from the impure solution (red lines) and the standard FI curcumin (blue lines); and (d) SAXS curves of spherulites (red lines) and FI curcumin (blue lines): orientation-average plot of the scattering intensity *vs* the modulus of the scaling vector.

Figure 4a shows the PXRD profile of the spherulites obtained from impure and pure solutions. For comparison, the simulated PXRD pattern of the FI curcumin (BINMEQ04) is also shown. The spherulite diffractograms are quite similar with respect to peak positions, regardless of being formed from pure or impure solutions. However, compared to the reported *P*2/*n* form of curcumin (BINMEQ04), there are some differences. For example, the peak at 2θ = 10.968° cannot be the suggested (1 0 −2) reflection because this reflection is absent in the *P*2/*n* space group. Therefore, the spherulite may be a new form of curcumin (perhaps a lower symmetry *P*2 or *P*1̅).

In Figure 4b, the Raman spectrum of the spherulitic material is compared with that of the FI curcumin needles. Figure 4b shows several spectral differences between spherulites and FI needles. The assignment of Raman bands was made based on the studies of López-Tobar *et al.*(23,24) For the case of spherulites, the intense bands at 1627 and 1601 cm^–1^, which can be attributed to ν(C=O)/ν(C=C) vibrations in the aromatic ring, respectively, undergo a huge shift (also, a peak split can be observed) and broadening, respectively. This change is usually related to conformational modifications on the side aromatic rings that cause a variation in the electronic conjugation degree along the entire molecule. The δ(C=C–H) weak band at 1318 cm^–1^ of the inter-ring chain undergoes a marked shift (1318 to 1312 cm^–1^). There is also a slight spectral change associated with the phenolic rings; the weak band at 1250 cm^–1^ undergoes a shift downward, and in spherulites, this peak contains a shoulder at 1236 cm^–1^. Based on the spectral differences, it is possible to generalize that curcumin conforms into a different crystal structure during the formation of spherulites that should be indifferent to the ones in the FI needle-shaped curcumin.

In Figure 4c, the DSC curve for the spherulites obtained from the impure solution is compared with that of the FI needle-shaped curcumin obtained from the pure solutions (lower supercooling) (Figure 4c). Spherulites exhibited a broad transition from 175 to 184 °C and a sharp transition at 182 °C, which is associated with a melting enthalpy of 36.87 kJ mol^–1^. This melting enthalpy is slightly higher than the melting enthalpy of FI curcumin measured in this study but matches with the melting enthalpy of FI crystals reported in the literature (37.19 kJ mol^–1^).^25^ One noteworthy feature is the appearance of a small melting endotherm at 183 °C for the spherulites. In the polymeric crystallization literature, this type of thermal behavior is frequently reported for spherulitic materials. The small endotherm peak is usually assigned as an annealing peak and is commonly attributed to the reorganization and remelting of the different layers of the lamellae.^26^ Unlike FI crystals, spherulites are more heterogeneous, and the melting behavior can slightly alter depending on the number of filaments, the thickness of the filaments, and, possibly, the length of the filaments. Another noteworthy observation is the absence of the glass transition region in the DSC curve of spherulites (not shown). In the polymer crystallization literature where spherulites are frequently reported, it is commonly assumed that the densely assembled fibers in spherulites might be connected *via* uncrystallized melts and impurities that cannot be probed with SEM or light microscopy.^27^ The unique melting endotherm with the melting point observed at 182 °C (see Figure 4c) without the presence of any glass transition confirms that spherulites are pure and stable and partly retain the thermal property of FI curcumin. This also means that the amorphous-like structural features (especially at the core of the spherulite) observed earlier in the SEM images (see Figure 3c) should be crystalline. DSC and PXRD both confirmed that spherulites are indeed crystalline. Typically, the assembling of multiple crystallites results in closely spaced dislocations with a wide range of burger vectors and can evolve into an amorphous-like spherulite core at the micron scale.^6^ However, in the present material, the spherulite cores seem to have retained long-range periodicity at the nanoscale. This type of observation was first reported by Penn and Banfield^6^ for inorganic materials, and this can be generalized now for the organic molecule curcumin crystallized from supercooled solutions. This also means that the crystallites are possibly connected *via* crystallite interpenetration rather than by uncrystallized zones or impurity phases.

To further explore the structural properties of the spherulites, SAXS analysis was performed on the spherulites obtained from impure solutions and compared against the SAXS data of the FI curcumin needles (Figure 4d). SAXS can provide structural information about the solid–solid interface that is frequently observed in the spherulites obtained from impure solutions. The SAXS curves of the two samples studied are different, clearly pointing to microstructural differences in these materials. In the case of the FI sample, a “typical” SAXS spectrum with a slope of about −3.6 is observed in the very low-*Q* range until *Q* ≈ 0.05 Å^–1^, whereas at larger *Q* values, the slope changes to −1.3. On the other hand, the spherulite sample gives SAXS spectra with a slope of −3.8, whereas at *Q* = 0.134 Å^–1^, a sharp Bragg peak is observed. The calculations of the interatomic spacing (*d*-spacing) for the observed Bragg peak at a *Q* of 0.134 Å^–1^ are equal to a characteristic size of *D*_Bragg_ = 47 Å. This *d*-spacing value could be attributed to a major microstructural characteristic of the material such as the crystallite unit cell or the average crystal–crystal distance. For fractal objects, according to Porod’s law, the scattered intensity *I*(*Q*) follows a power law, *I*(*Q*) ∼ *Q*^–*a*^, where *a* is a fractal dimension. For mass fractals, 1 < *a* < 3, while for surface fractals (scattering from surfaces), 3 < *a* < 4. The SAXS profiles of both solid structures follow a power law—[*I*(*Q*) ∝ *Q*^–*a*^]. Bale and Schmidt^28^ derived *a* = 6 – *D*_s_ for a rough interface with a surface fractal dimension of 2 < *D*_s_ < 3. For a smooth surface, *a* = 4 and *D*_s_ = 2 (Porod’s law^29^) have been predicted. The slopes of the scattering curves of FI and spherulites are clearly different. The FI sample produced SAXS spectra with two slopes with the values of *D*_s_ equal to 2.4 and 4.7. The SAXS spectra following a power law of *Q*^–3.6^ signify the scattering from rough interfaces, whereas the regions following a power law of *Q*^–1.3^ indicate the existence of an open fractal structure. The spectral transition from *Q*^–3.6^ to *Q*^–1.3^ indicates that the scattering from the local structure of the FI curcumin needles is dominated by the arrangement of fractal structures at length scales of 12 nm and below.^30^ The correlation length ξ for the FI sample was calculated from the two regions of SAXS spectra with different slopes and was found to be 240 and 70 Å. The correlation length ξ represents the characteristic distance above which the mass distribution is no longer described by a fractal law. This value for spherulite was found to be 110 Å. These numbers could be attributed to the size of the formatted crystallites, fractal space due to lattice defects, or characteristic sizes between the formatted regions. The size of *D*_Bragg_ ≈ 50 Å, obtained from the Bragg peak, which is observed only in the spherulite matches with the values reported by Schliehe *et al.* during the formation of two-dimensional (2D) PbS sheets *via* particle attachment mechanisms.^31^ This size can be the fractal space that could exist between the crystallites. The calculated correlation lengths of both FI and spherulite samples also match with the sizes reported for self-assembled fructose–curcumin structures with different morphological features.^32^ Finally, it is worth highlighting here that the crystallite sizes obtained from SAXS profiles are 1–2 orders of magnitude smaller than the crystallite size observed from SEM/TEM. This can be attributed to the thickening and coarsening of agglomerated crystallites (this can be experimentally observed in SEM images shown earlier in Figure 3a–d). Typically, aggregation occurs at the finer scales than the ones shown in Figure 3,^6^ which could be the reason that the larger sized crystallites observed in SEM/TEM (at the studied resolution) are larger than the crystallite diameter obtained from SAXS.

### 2.4 Pre-nucleation Stage and Mesophase Analysis

Classical crystallization *via* molecule-by-molecule addition eventually results in the formation of single crystals and more often retains the crystal equilibrium habit.^8^ Microscopic analysis clearly showed the spherulitic structure of the final crystals with several interesting morphological features, not resembling any equilibrium habit of the curcumin crystals. In particular, spherulites showed and contained several nanometer-thick crystallites and solid–solid interfaces. Solid–solid interfaces are a feature of crystallization by nonclassical mechanisms that usually involve particle aggregation or coalesced growth of two crystallites.^33,34^ Alternatively, a solid–solid interface can be obtained due to the attachment of a liquid-like condensed mesophase from the solution followed by its coalesced growth.

**Figure 5:**
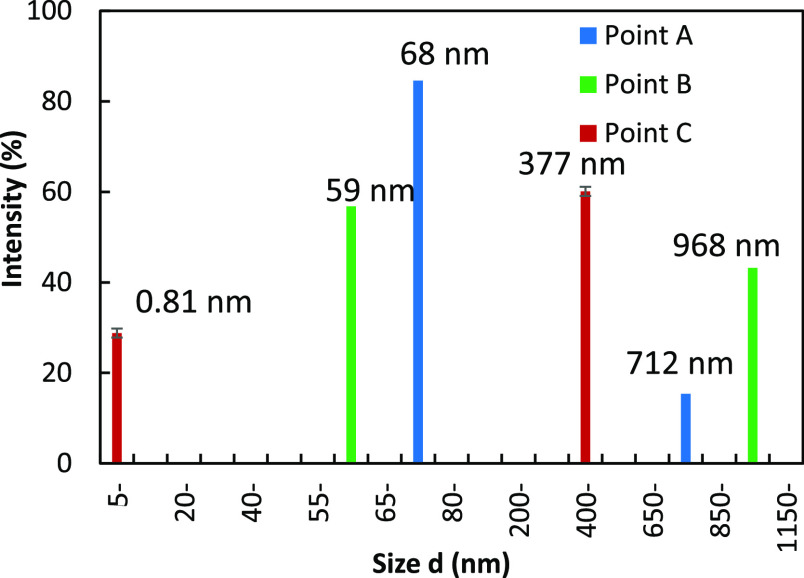
Histogram showing the hydrodynamic diameter of the particles measured using DLS. The values observed may correspond to single molecules, crystallized or pre-crystallized units (intermediate phase; the values of the actual measured sizes are also shown above the bars) in the highly supercooled solution during three different stages of the crystallization of curcumin into spherulites (point A: before nucleation, point B: during crystallization, and point C: after nucleation or in the saturated solution).

In Figure 5, dynamic light scattering (DLS) results are given, where point A is before nucleation, point B is during nucleation, and point C is after nucleation. The measurements show the presence of “bodies” in sizes ranging from the size of one molecule (0.81 nm) to ∼960 nm depending on the experimental conditions. DLS results confirm the presence of two types of bodies that differ by size (average solvodynamic diameter of 68 and 716 nm) prior to nucleation and also during the course of nucleation (average diameter of ∼59 and ∼968 nm). After nucleation (point C), the solution seems to contain only one type of particle with an average hydrodynamic diameter of 377 nm with the remainder being the single molecules of curcumin with an average hydrodynamic diameter of ∼0.81 nm. This (point C) may be a property of the saturated solution; the saturated solution contains particles with sizes that are several nm larger than the size of a unit solute molecule.^35^ The presence of two peaks (two discrete sizes) during all stages of measurement reveals that the smaller sized particles are present in surplus quantities, such that their Brownian motion can be exclusively discriminated from the larger particles and their sizes can be predicted. The difference in particle size results obtained from DLS measurements and with the microscopic techniques shown earlier is due to the differences in the weighted averages determined in each case and differences in the physical property that is measured (solvodynamic area *vs* projected area). These experimental observations confirm the existence of an intermediate phase, in addition to the single molecules of the curcumin, and some of them are almost equivalent to the size of the filaments observed in the mesocrystals. This intermediate phase can integrate with the existing crystallites without any specific crystallographic orientation and thicken the size of crystallites or could evolve into individual crystallites. This usually results in superstructures (like spherulites).^3,7,36^ Additionally, for particle aggregation to proceed, it is necessary to have such an intermediate phase in the solution of right size. If the growth units are smaller, they will dissolve in the solution, and if they are larger, particle attachment will be harder.^37,38^ At least for the case of the curcumin/IPA system, this is typically less than a micron. These interfaces may either dock on a pre-crystallized unit or form into a primary crystallite. These crystallites can merge to form a superstructure *via* oriented particle attachment (this is detailed in the Discussion section).

**Figure 6:**
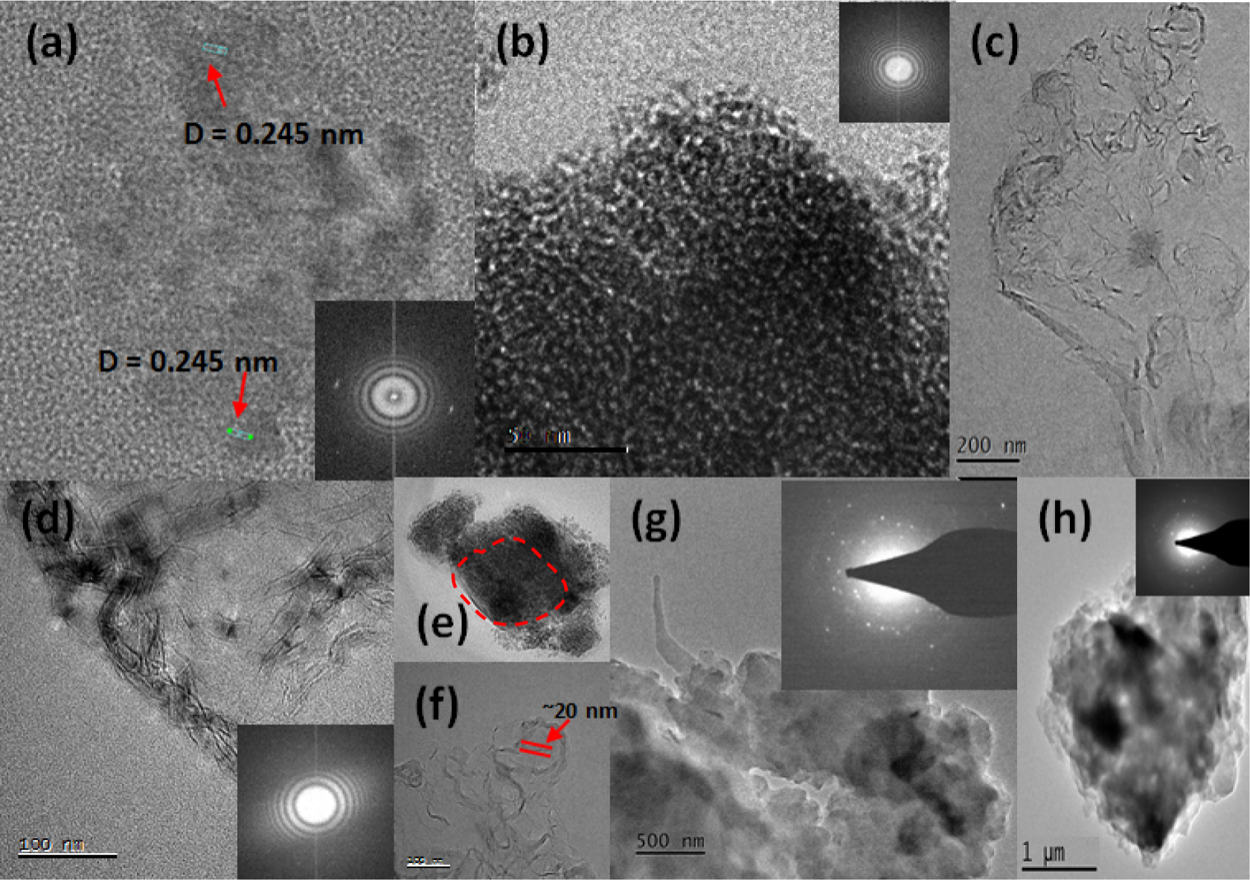
TEM images showing the different possible structures of the intermediate phase that exists in the supercooled impure solution roughly 1 h before the point of nucleation. The SAED pattern shows that these intermediate phases are crystalline or amorphous or a combination of both. (a,f) Lattice lines and *d*-spacings of the crystallite observed on the TEM grid—clearly, these are crystalline structures. (b) Dense structure indicative of a pre-nucleation cluster. (c,d) Some featureless structures with a ribbon-like topology. (e) Boundary of the plate-like structure with facet edges emerging from an amorphous-like cluster with red dotted lines. (g,h) Featureless structures; however, they are crystalline (see the SAED pattern). The figure shows that the intermediate structures that are present in the solution may be either crystalline or amorphous with different morphologies that may not represent each other.

TEM images (Figure 6) show the presence of a wide range of particles that may be crystalline, amorphous, or a combination of both and that their sizes span between 50 nm and a few microns. These intermediate phases do not exhibit a unique pattern; rather, they show a wide range of nanostructural features that include plates, ribbons, micron-sized 2D sheets, or featureless structures. SAED pattern analysis showed that these intermediate structures are either crystalline or amorphous. TEM images also showed evidence for the formation of facetted crystals from randomly arranged crystallites: (i) Figure 6e captures one of the mechanisms in the formation of plate-like crystals; the randomly attached crystallites are recrystallizing into a plate-like crystal (evidenced by the crystal facets) and (ii) Figure 6c,d clearly shows the appearance of lattice lines randomly twisted, forming nanoribbons (∼20 nm thick). This combination of observations at least confirms the existence of intermediate phases in the solution that precedes the formation of a stable solid phase.

The different nanostructural features of the intermediate phase also reveal the fact that the spherulite formation mechanism may be a more complex process. It should be acknowledged that the TEM grids were prepared following a rudimentary approach that relies on flash evaporation to deposit the intermediate solid structures at the pre-nucleation stage (please see the Experimental Section). This approach has several practical limitations related to capturing the structures that appear in the solution and to avoid artifacts generated by the sample preparation. Recently, Cookman *et al.*(39) used liquid cell TEM and observed several structurally featureless structures (similar to the ones observed in Figure 6) while capturing the nucleation of an organic compound from its dilute solutions under electron beam. They showed that the less-stable intermediate structures attach to the facets of the single crystals, and eventually, the single crystals grow at the expense of these intermediate phases in the solution.^39^ Despite the crude approach adapted in this work, we found that the TEM images are useful and were earlier used to expose the nonclassical growth behavior of organic crystals, especially to have a rough idea about the possible structure of intermediate phases.^40^

### 2.5 Anomalous Growth of Spherulites *via* Growth Front Nucleation

**Figure 7:**
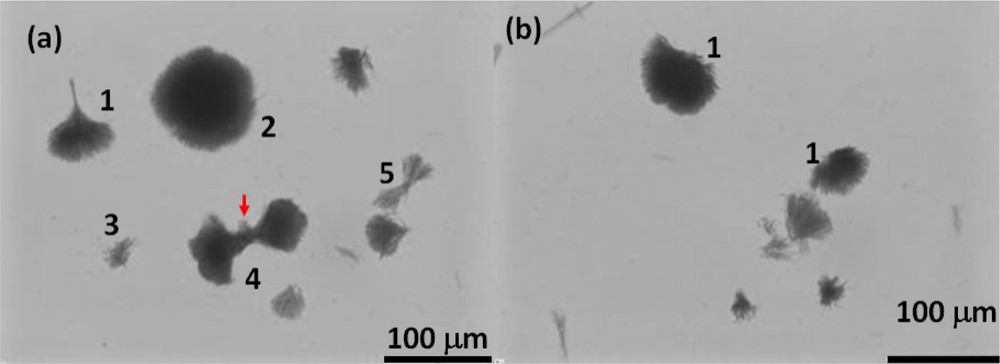
(a,b) Light microscopic images showing the anomalous growth pattern of spherulites: crystals are collected from the highly impure solution after 5 min from the point of nucleation [**1**: fan-shaped crystals, **2**: fully developed spherulites, **3**: featureless structure, **4** and **5**: bowtie structure with loosely (or less-dense) assembled filaments]. Note that both (a,b) are two different snapshots of the same sample.

The growth pattern of mesocrystals is anomalous and is difficult to predict due to the lack of specific growing facets, the heterogeneous nature of surfaces, and the overall morphology. In the literature, growth mechanisms of mesocrystals are mostly proposed based on the final structure.^1,7,41^ To identify the formation mechanism, liquid samples were collected from the crystallizer 5 min after the point of nucleation, and the habits of the solids in the solution were analyzed using a light microscope (see Figure 7). Samples collected at this stage could provide some information about the steps involved in the spherulite formation. Figure 7 shows some unique morphological features; light microscopic images showed fully developed spherulites (**2** in Figure 7) and unfinished spherulites with different shapes (**1**, **3–5** in Figure 7) that resemble fan (**1**), bowtie (**4**, **5**), completely developed spherulite (**2**), and a featureless structure (**3**). These structures can be formed if the mesocrystals tend to form *via growth front nucleation* and low-angle branching from a common point.^42^ The term “growth front nucleation” refers to the repeated secondary nucleation (see also Section 3) at the crystal growth front.^43^ Growth front nucleation together with low-angle branching is only reported for the crystallization of melts^10^ and now for an organic molecule from its solution.

The spherulites might have formed *via* the growth of several thread-like fibers in one specific direction (the fiber-like structures can be seen in the SEM images shown earlier in Figure 3a–d) that originate from either one end (see **1** in Figure 7) or from both the ends (see **4** and **5** in Figure 7) of the growth front, resulting in fan-shaped and bowtie-shaped structures, respectively. Additionally, light microscopy images showed the appearance of a new growth front located in between the ends of two growth fronts (see the red arrow in **4** of Figure 7), where possibly, several new fibers can stretch out *via* low-angle branching. This growth pattern is observed in nature during mineralization and urinary stone formation,^44,45^ polymer crystallization,^46^ and now for the first time formed naturally for an organic molecule during its growth in impure solutions. In the case of pure solutions, a similar growth pattern (not shown) was observed in the solid samples collected from the crystallizer at 5 min after the point of nucleation.

## 3 Discussion

### 3.1 Possible Mechanisms for the Formation of Pure Spherulites from Impure and Pure Solutions

**Figure 8:**
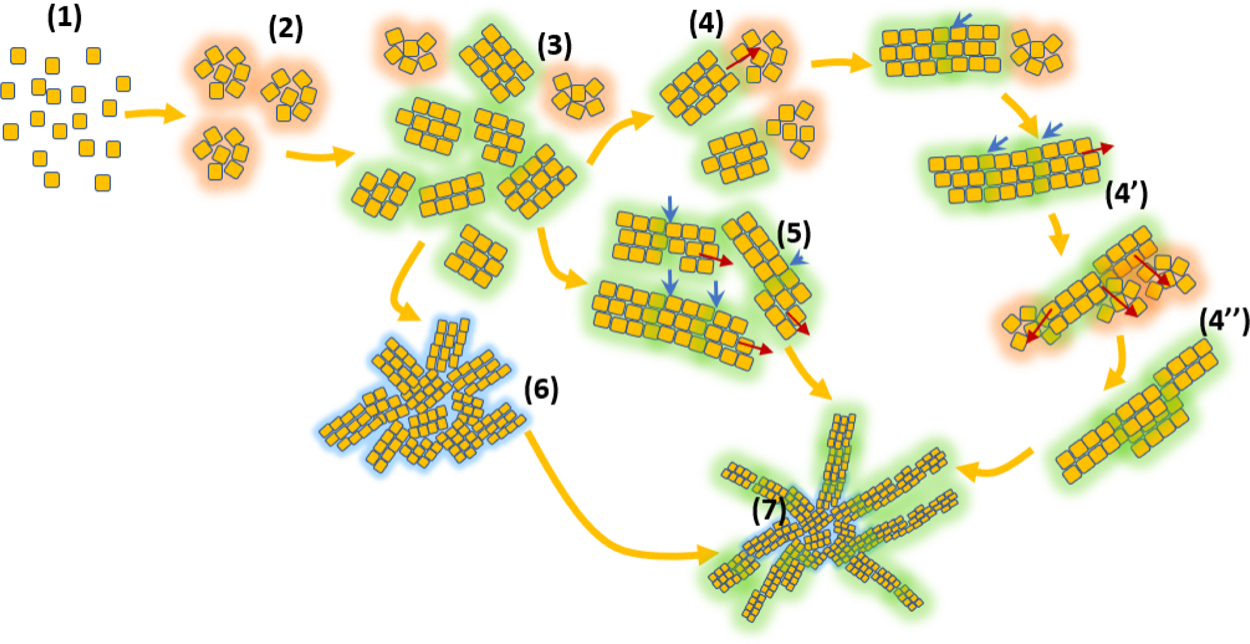
Possible mechanisms involved during the formation of curcumin spherulites. (**1**) Molecules in the liquid phase, (**2**) formation of pre-nucleation clusters (glowing red), which eventually evolve into a stable crystallite (**3**); this metastable pre-nucleation cluster might go through a structural reorganization, forming a mesophase, which eventually evolves into a crystallite (glowing green), (**3**) several nm-sized stable primary crystallites and several intermediate or mesophase structures; we call them as intermediate structures (glowing red) or a mesophase to differentiate it from the pre-nucleation clusters, as these structures are in the solution that already contains a stable solid phase (these structures can simply be called as nucleation clusters, as they contribute to the repeated 2D nucleation), (**4**) attachment of the nucleation clusters onto the already formed stable crystallite, and (**4–4′**) one-dimensional stretching of crystallite *via* formation of a new stable crystallite from the mesophase attached already on the primary crystallites. This process represents the growth *via* repeated 2D nucleation, and the repeated 2D nucleation occurs on the growth front, which leads to the stretching of the filaments in the spherulites, (**5**) perfectly ordered and (**6′**) imperfect alignment of nanometer sized crystallites (glowing blue); both of these structures can further evolve *via* a coalesced growth, (**4–4′** and **3–5**) growth of spherulite filaments *via* combination of particle attachment and repeated 2D nucleation, (**4″**) possible growth and thickening of primary nanocrystallites *via* repeated 2D nucleation. (The red arrows show the direction of the growth front or the location where a new growth front nucleation can possibly occur. The blue arrows show the solid–solid interface.)

Observations from chromatographic, thermogravimetric, and microscopic analysis clearly showed that spherulites obtained from an impure solution are highly pure (>99%) and exhibit good thermal stability, crystallinity, the presence of several solid–solid interfaces and crystallite docking points, perfectly or imperfectly oriented crystallites, and a crystallographic hierarchy. Observations from DLS experiments and TEM confirmed that the spherulite formation was preceded by the existence of an intermediate or mesophase. Based on these observations, it is possible to hypothesize different possible mechanisms for the formation of pure spherulites: (i) spherulite growth *via* particle aggregation (**5** and **6** in Figure 8) and (ii) spherulite growth *via* repeated surface nucleation (**4–4′** in Figure 8) at the growth front (see the red arrows in **4** and **4′** of Figure 8, showing the direction of the growth front). The first one was proposed based on the observed crystallite docking in microscopic images and the existence of intermediate or mesophases and multiple solid–solid interfaces in the spherulites. The second one relies on the concept of repeated secondary nucleation, also known as double nucleation,^47^ at the growth front and the existence of the fine-scale polycrystallinity in the microscopic images of spherulites.

For growth *via* repeated 2D nucleation, the metastable or intermediate phase in the solution can evolve into individual crystallites (**2–3** in Figure 8), or they can attach to the already formed stable crystallite and evolve into a new stable crystallite (**3–4′** in Figure 8); this step usually results in the filament-like structure, as observed in the spherulite. Similarly, a stable crystallite can aggregate in a more random way (**6**) or in an ordered (**5**) fashion to form a superstructure, like spherulite. The crystallites formed at the end of steps (**3**), (**4**), (**4′**), (**5**), and (**6**) can involve in particle aggregation to form spherulites (**7**). Such growth behavior was reported during the precipitation of alloys.^48,49^ Growth *via* any of these mechanisms usually results in superstructures with a crystallographic hierarchy similar to the spherulites obtained in this work.^42,43^ This type of growth behavior involves the elimination of some solid surfaces and the creation of new solid–solid interfaces. The particle aggregation mechanism was proposed based on the existence of several randomly arranged crystallites (see the SEM image in Figure 3c) at the core of the spherulites (especially in the spherulites obtained from the impure solution) and a slightly ordered arrangement of crystallites (see the SEM image in Figure 3d) in the filaments. At the early stage, the solution is enriched with a large number of primary crystallites and probably with lots of line defects with a wide range of binding energies. Crystallites with lots of defects also mean that the surface is more heterogeneous, and this favors the random attachment of crystallites; the system will try to reduce the surface energy by fast elimination of every possible interface with highest binding energy rather than a specific surface of the primary crystallites. Crystallites with line defects can merge together to form the disordered, yet crystalline, core with different forces that may be different from the forces involved when two crystallites try to achieve full coordination. The formation of larger sized crystallites *via* assembly of primary nanocrystallites is earlier observed with inorganic materials.^33^ Theoretical studies performed earlier by other researchers with metals show that two crystallites can naturally align themselves to mechanically relax the highly stressed interface that formed upon contact.^50^ Despite the cohesion energy involved during the crystallization of organic molecules, this will be much lower than in metals, and it is still possible to expect such a phenomenon to occur (as evidenced by the lack of any common crystallographic orientation). Formation of the spherulite core *via* the random arrangement of crystallites can also be expected if the process is entropically controlled (as discussed earlier in Section 2.1). Once the supersaturation is slightly consumed, the growth should occur *via* a combination of entropically and chemically controlled processes. At this stage, if the crystallites contain fewer line defects, such crystallites contain essentially homotattic surfaces. These crystallites will eliminate a specific facet with high binding energy, or in other words, crystallites will fuse in a specific direction by eliminating a specific interface. This argument is valid, if we look into the morphological features, including the ordered docking of crystallites and multiple crystallite docking points on the filaments in the spherulites obtained from impure solutions. It is more likely that the ordered docking of crystallites should have occurred at a scale much lower than the scales shown in the SEM images (Figure 3b–d). These ordered docking processes can reflect the coalesced growth of two docked crystallites, which can be observed under SEM. Crystallite fusion is commonly observed during crystallization from the amorphous phase of inorganic molecules, where fewer atoms are involved. For instance, Schliehe *et al.* recently reported on the 2D growth of an approximately half-micron-sized inorganic PbS material *via* ordered self-assembly of several nanocrystallites (5 nm) with *in situ* TEM.^31^ Addition of nanocrystals followed by fusion of those particles was also reported during the formation of titania crystals and other inorganic materials.^31,51^

Alternatively, spherulite can also evolve *via* the earlier mentioned repeated 2D nucleation or “double nucleation”, as typically referred to in the biological literature. The repeated 2D nucleation refers to the tendency of the critical nucleus to repeatedly attach on the growth front (**4** and **4′**) that can evolve into a fiber-like polycrystalline structure. Double nucleation refers to a process where a crystalline material evolves through two different pathways that occur simultaneously. First, the nucleation occurs in the bulk solution *via* homogeneous nucleation (**1–3**), followed by a second step where the nucleation clusters or intermediate phase (**4** and **4′**) assembles on the surface of the pre-existing stable nuclei (the mechanism **2–4** and **7′** can occur in parallel). Repeated 2D nucleation can also occur on the other face of the growing filament (**4″**), which will lead to thickening of the spherulite filaments. This argument is made based on the DLS and TEM results, which confirm the existence of some intermediate phase prior to nucleation and even after the point of nucleation. It is presumed that these pre-nucleation structures preferentially settle on the growth front, leading to the growth of the filaments in the spherulites. In the polymeric crystallization literature, the repeated secondary nucleation mechanism is considered to be an essential feature during the formation of polycrystalline spherulites.^42^ In fact, SEM images of the curcumin spherulites support this theory based on the observed fine-scale polycrystallinity, which appears to be anisotropic at the micron scale (see the SEM images in Figure 3a–d). The existence of double nucleation can be realized from Figure 7, where fully developed spherulites and incomplete spherulites coexist. This means that both structures are being formed within the solution at the same time. The concept of double nucleation is not fully hypothetical, and in fact, a recent study performed using the state-of-art liquid cell TEM but with dilute solutions confirmed that such mechanisms seem to drive the growth of a single crystal during the homogeneous nucleation of flufenamic acid from its solution.^39^

The particle aggregation mechanism in Figure 8 was proposed based on the SEM and TEM images, which showed the existence of a solid–solid interface and multiple crystallite docking points. During the growth of crystals in impure solutions, at the core of the spherulite, we observed nanometer (∼200 nm)–near-micron-sized crystallites assembled or docked imperfectly at the core and a more ordered arrangement of crystallites at the filaments that stretch out from the core. Such a superstructure should have evolved *via* the attachment of the crystallites, where one facet of a crystallite will dock with a particular facet of another crystallite and merge to form the superstructure (spherulites). TEM images confirmed the coalesced growth of two crystallites and the existence of interference patterns at the micron scale. The interference pattern (see Figure 3l–r) is also evidence for the crystallite thickening *via* stacking and docking of crystallites.

It is worth highlighting that according to the Ostwald rule of stages, larger crystals grow at the expense of smaller crystals. Spherulites exhibit a crystallographic hierarchy, where crystallites of different sizes coexist in the superstructure (discussed earlier based on Figure 3a–h). This type of structural evolution is possible only if the thicker filaments show some sympathy so that the new crystallites with smaller size grow on the already grown surface, leading to filament branching (for instance, a particular branch can elongate indefinitely instead of the formation of a new branch which ultimately fills the space of the spherulite). This particular phenomenon, which is commonly referred to as sympathetic nucleation, occurs when new smaller sized crystals can grow at the sympathy of larger sized crystals. In the case of spherulites, a new filament grows on the developing spherulite at the mercy of the larger sized filaments. This is clear from the light microscopic image, where a new growth front evolves in the middle of the bowtie-shaped structure (see Figure 7 and Section 2.5). This type of growth is commonly observed during the precipitation of solids, and this is the first time we observe during the crystallization of organic compounds from their solution.^48^

Typically, spherulites occurring naturally in biological and geological environments are assumed to grow due to the presence of impurities.^44^ However, for the case of curcumin, both pure and impure solutions produced spherulites under the studied experimental conditions. In fact, not many systems grow as spherulites without impurities.^52^ Curcumin seems to be the only system reported so far to form spherulites from its pure solution without the presence of any additives. The anomalous growth behavior of curcumin was not completely new for the curcumin system, and formation of superstructures (not spherulites) was earlier encountered during the precipitation of this compound from its bulk solution or in the presence of a surfactant; the only difference is that the fiber-like structure emerges out at the tip of needle- or rod-shaped crystal seeds and spreads mostly in *x*- and *y*-directions rather than in the *xyz*-directions.^53^ Observation of clustered needles was also reported during the evaporative crystallization of this molecule from ethanol and also during the precipitation of curcumin in ethanol in the presence of anti-solvent water.^40^ It should be mentioned here that none of these studies show any evidence that reflects the properties of mesocrystals or sign of crystallographic heterogeneity and purity. Additionally, none of these studies report on the experimental conditions that drive the crystallization toward the nonclassical routes or discuss the effect of the impurities on the morphology and purity of the final crystals and the solid formation mechanisms in pure and impure solutions. In the present study, based on the present experimental conditions, the formation of spherulites can be attributed to the degree of supercooling. This was confirmed by performing several additional batch experiments at 5 °C but with lower levels of supercooling ranging at Δ*T* = 40–20 °C (not shown in this work), and all of these experiments always produced needle-shaped single crystals. In fact, the literature reports show that spherulites are mostly encountered in supercooled fluids, colloidal glasses, and highly undercooled viscous melts.^52,54,55^ Supercooled solutions contain long-lived dynamic heterogeneities (a situation when some molecules move orders of magnitudes faster than those situated only nanometers away) and cooperative molecular movements (if one molecule moves, another molecule moves closely following the first).^56^ The dynamic heterogeneity can alter the shear viscosity and the translational and rotational diffusion coefficients. The ratio of rotational and translational diffusion coefficients decreases by orders of magnitude, and the molecules will translate at larger distances before they rotationally decorrelate from their initial orientation.^42,43^ Both of these properties favor the formation of misoriented or imperfectly oriented crystallites *via* nonclassical crystallization, in particular, repeated 2D nucleation at the growth front.^42,43^ If the rate of molecular reorientation is slower than the rate at which the growth front grows, the formation of misoriented crystallites is more likely. Likewise, if the rate of molecular reorientation at the growth front is faster than the rate at which solidification occurs at the growth front, it is more likely to see a more ordered crystal. Although the impurity molecules are not incorporated into the crystals, if they can disturb the rate of molecular reorientation at the growth front, it can contribute to misorientation at the growth front. This particular concept can be used to explain the morphological differences observed at nano- to micron scale in the spherulites obtained from impure (see Figure 3a–d) and pure (see Figure 3e–h) solutions. Additionally, if the solidification of curcumin at the growth front occurs at a pace faster than the time required for an impurity molecule to be recognized by the location on the growth front for integration, eventually, the crystallized materials can be expected to be pure. Adsorption theory supports this argument; the higher the number of adsorption sites, the higher the chance of adsorption and the lower the activation energy of the surface diffusion of the molecules on a substrate.^57,58^ This means that impurities that contain fewer interacting sites are less likely to be rapidly recognized in the crystal lattice or strongly adsorbed at the growth front.

Irrespective of the spherulite formation mechanism, either purely *via* particle attachment or *via* repeated 2D nucleation, the HPLC results shown earlier confirmed that the final product remains pure. This can be explained based on the time scale involved during the formation of mesocrystals and their single-crystal versions. In Figures 1a and 2a, the rate of crystallization was estimated based on the slope of the mass of the crystalline material with time (g L^–1^ min^–1^). The rate of spherulite crystallization into a pure solid phase from the highly supercooled solution was roughly 41% faster than that of the spherulite crystallization kinetics observed in its pure solution. Based on this particular observation, it is possible to propose that the molecules tend to assemble so fast to form the primary crystallites such that impurity molecules do not have enough time to reside and settle within the pre-nucleation clusters or assemble within the crystal lattice. The impurity retention time is difficult to predict experimentally, especially at the pre-nucleation stage or even during the crystallization; however, there are few case studies from the crystal growth literature where the time of impurity adsorption was predicted during crystal growth using a semi-empirical expression that supports this theoretical hypothesis.^4^ For instance, in the studies of Guzman *et al.*(61) and Kubota *et al.*,^59^ impurities are barely incorporated into the growing crystal when impurities are slowly adsorbed onto the growing faces. In the present case, the spherulites are formed in the impure solution with a pace that is roughly 40% of the crystallization rate observed in the pure solution, which seems to be sufficient to control the impurity transfer in the final product. In other words, this rate might be faster than the time required to reach the adsorption equilibrium or the retention time required for an impurity molecule to stay and integrate into the crystalline product.

The purity of the final crystals can also be attributed to the growth of spherulites *via* repeated 2D nucleation. Typically, during crystallization, after nucleation, the crystals tend to grow *via* a molecular attachment process, where the final crystal will usually retain the equilibrium crystal habit. However, during the growth process, it is more likely that structurally similar impurities can replace the crystal lattice. On the other hand, nucleation usually produces pure solid forms. For the case of spherulites, as the structure evolves *via* a combination of particle attachment and repeated 2D nucleation, we propose that (at least for the studied system) these mechanisms prohibit the incorporation of the structurally similar impurities into the final crystals.

In Figure 8, we generalized the two concepts to explain why curcumin precipitates into pure mesocrystals or impure single crystals from its impure solutions. The formation of single crystals is usually associated with the classical mechanism of molecule-by-molecule addition, where a pre-nucleation cluster evolves into primary crystallites followed by their growth into a single crystal. On the other hand, as illustrated in Figure 8, the mesocrystals are formed *via* different mechanisms that involve the (i) formation of nanocrystallites, followed by repeated 2D nucleation or double nucleation, and (ii) reduction of surface energy *via* particle attachment. If the time scales involved in both of these cases are similar, the concept of impurity retention time can play a significant role in the purity of the final product. During the mesocrystal formation, if the impurity retention time on the crystallite surface will be slower than the rest of the process, the resulting mesocrystal should be essentially pure.

**Figure 9:**
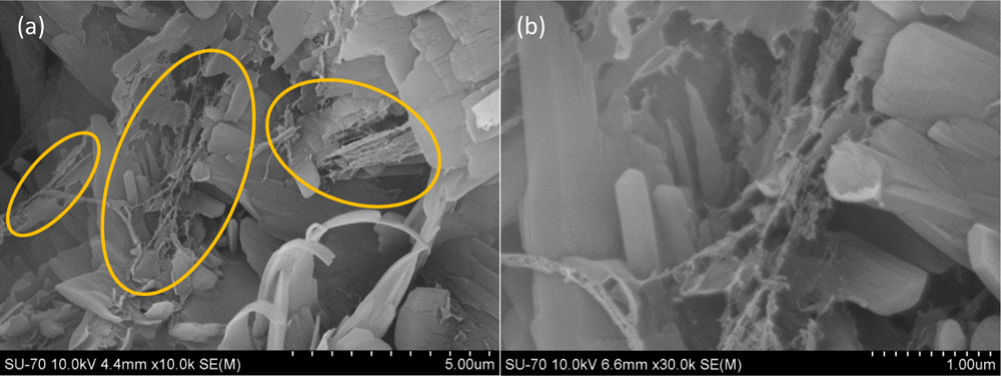
(a) SEM image showing the core of a spherulite (circles showing the net-like topology) and (b) magnified image of the spherulite core exposing possibly the early structures of the branching filaments that stretch out from the spherulite core.

Apart from the discussed spherulite formation mechanism, the existence of several branchings separated by a low angle is difficult to explain based on the morphological observations or from the measured kinetics. Spherulite crystallization theories state that radial branching is a property of the spherulites, and branching occurs at a fixed non-crystallographic axis.^4,60^ Close inspection of the spherulite core shows that the branching involves an even more complex step. Branching seems to precede from a net-like structure (see the circled areas in Figure 9a) composed of fibers of approximately 10–20 nm thickness and roughly 5 μm length (see Figure 9b); they are also separated from each other by a few nm. Based on the final structure, it can be inferred that these nanometer-thick fibers should have emerged from the core (essentially the nuclei, which cannot be probed at this resolution) of the spherulite *via* the earlier discussed repeated 2D nucleation. The images in Figure 8 are obtained from the spherulites grown in impure solutions, and such features are not observed in spherulites (not shown) obtained from pure solutions. It is probably the case that in the pure solution, the branching of the crystalline fibers might have followed a different mechanism, or such a mechanism should have occurred even at finer scales. The finer scales of the fibers observed in the microscope can also be related to the high level of purity of the spherulites obtained from the impure solution. In general, during crystallization, it is more likely that incorporation of impurities mostly occurs during the growth of the crystals after the point of nucleation *via* adsorption. In the case of spherulites, the structure seems to evolve through the nonclassical route from these fiber-like structures *via* the earlier proposed repeated 2D nucleation. As these structures reject the impurities during the evolution of spherulites, the final structure should remain pure.

## 4 Conclusions

For the first time, the formation of spherulites has been identified during the crystallization of an industrially important organic compound, curcumin, from its pure and impure solutions, the latter containing two structurally similar impurities. The spherulites are formed in a process operated with high supercooling, that is, high supersaturation and low crystallization temperature. From the highly impure solution prepared from the commercially available curcumin (purity of about 72%), the solid product receives high purity after a single crystallization step *versus* the four consecutive crystallization steps that are required to obtain the same level of purity *via* classical crystallization. Characterization of the mesocrystals allowed us to hypothesize that the formation of spherulites is driven by nonclassical crystallization pathways that involve particle aggregation and double nucleation. The results clearly show that a nonclassical crystallization pathway can offer a new route for the purification of organic compounds. Spherulites are often considered as unwanted crystallization, as they are often preceded by complex and multiple mechanisms that cannot be explained with classical theories. On the other hand, spherulites possess several interesting properties. First, their surfaces reject impurities during their formation in impure solutions, and they contain an enormous external surface area and self-regulate their final product size. Finally, if the concept of obtaining pure mesocrystals from impure solutions with supercooled solutions holds for other systems, it is possible to propose a new experimental design for the purification of organic compounds. For instance, an impurity concentration can be artificially spiked to delay the induction time in supercooled solutions and find the experimental window where the system can be forced to nucleate *via* a nonclassical pathway that might be free of impurities.

## 5 Experimental Section

### 5.1 Materials

Commercially available crude CUR was purchased from Merck (CUR > 75% nominal purity; HPLC, area %; containing 99.9%), and HPLC-grade IPA (>99.9%) was purchased from Sigma-Aldrich and used without further purification. HPLC analysis performed in our laboratory confirmed that the as-received product contains 78.6 wt % CUR, 17.8 wt % DMC, and 3.6 wt % BDMC; the protocol used for the HPLC analysis is described elsewhere.^15,17,18^

### 5.2 Crystallization Experiments

All the crystallization experiments were performed in the batch mode in a 100 mL EasyMax synthesis workstation at the working temperature; a reactor volume of 100 mL was used for all the experiments. Agitation was provided using an overhead (with a pitched blade) stirrer. The temperature inside the crystallizer was maintained using an external jacket that relies on electrical heating and solid-state cooling technology. The agitation inside the crystallizer in all the experiments was maintained at 250 rpm.

Pure or crude curcumin is dissolved in IPA corresponding to a solution being approximately saturated at 60 °C. All the solids are dissolved by heating the solution to 70 °C for 30 min. For the production of spherulites, the solution was cooled down to 5 °C (experiment 1) at a cooling rate of 8 °C min^–1^. This generates a supercooled solution of Δ*T* = T* – *T*_w_ ∼ 55 °C or *S* ≈ 6.20. The supersaturation was defined in terms of the ratio of the initial concentration of curcumin in the solution to the solubility concentration at the working temperature, *S* = *c*/c*. In all the crystallization experiments, we maintained the solution at the working temperature for ∼24–72 h depending on the experimental conditions to achieve complete saturation after nucleation. The rapid cooling rate was chosen in all the experiments only to maintain the experimental consistency. In all the crystallization experiments, the solid concentration or the suspension density was monitored using *in situ* Raman spectroscopy.

The solution for crystallization of curcumin spherulites from the impure solution was prepared by dissolving 0.6 g of crude curcumin in 100 mL of IPA. With respect to the composition of the crude solid phase, this generated a curcumin concentration of 4.716 g L^–1^, demethoxycurcumin concentration of 1.068 g L^–1^, and bisdemethoxycurcumin concentration of 0.216 g L^–1^. The curcumin concentration is slightly lower than the solubility of curcumin (4.8 g L^–1^) in IPA at 60 °C. The solution for crystallization of curcumin spherulites from the pure solution was achieved by dissolving 0.471 g of pure curcumin in 100 mL of IPA solvent. For crystallization of FI curcumin needles from pure and impure solutions, we followed the same procedure, and the only difference is that the solution was cooled to 25 °C instead of 5 °C.

### 5.3 Impurity Profiling

The depletion in the concentration during the crystallization of curcumin in impure solutions was monitored in the offline mode using HPLC. Samples were taken at different time intervals during crystallization using a 1 mL syringe. The samples were then filtered using a 0.2 μm poly(tetrafluoroethylene) filter, and the concentration of the impurities in the filtrate was quantified (HPLC Agilent Technologies 1260 Infinity Series) using the procedure reported elsewhere.^15^

### 5.4 Determination of Solid Concentration Using Raman Spectroscopy

The concentration of the solids in the crystallizer was monitored using *in situ* Raman spectroscopy. Raman spectra were collected using a RXB1 Raman spectrometer from Kaiser Optical Systems, Inc. (Ann Arbor, MI, USA). Backscattered radiation was collected from the sample using a 1/4 in. immersion probe, which is coupled to the spectrometer *via* a fiber-optic cable. The power at the sample is approximately >150 mW. The probe was immersed in the crystallizer (positioned roughly 2 cm above the vessel base). iC Raman software (Mettler Toledo) was used to monitor and collect the spectra; the measurement region is 150–3425 cm^–1^ at 786 nm excitation, and the spectral resolution is 4 cm^–1^.

Several trial experiments performed at different temperatures show that the Raman intensity increases linearly with the solid concentration in a completely saturated solution. Based on this, a calibration-free method was used to correlate the Raman intensity to the mass of curcumin crystallized in the solution. This calibration-free method relates the Raman intensity with the consumption of supersaturation, Δ*C*, by the following expression
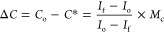
1
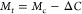
2where Δ*C* = *C* – C* at any instant of time (g L^–1^), *M*_c_ (g L^–1^) is the mass that can be crystallized, which can be obtained from a simple mass balance based on the initial experimental conditions and solubility at the studied temperature, *I*_o_ is the intensity of the Raman peak that corresponds to the initial concentration of the completely dissolved solution, *I*_*t*_ is the intensity of the Raman peak at any instant of time during crystallization, and *I*_f_ is the intensity of the Raman peak after complete saturation due to the crystal growth. This expression agrees with the Beer–Lambert law that the relationship between the incident and transmitted radiation intensities in vibrational spectroscopy changes linearly with respect to the sample concentration. Raman spectra were collected at a laser intensity of 400 mW in the −800 to +3600 cm^–1^ range with a resolution of 4 cm^–1^ and were averaged over 30 scans using an exposure time of 2 s. Despite the simplicity of this expression, it is independent of the effect of exposure time, position of the probes inside the crystallizer, laser power, and hydrodynamics of the vessel, which can be altered due to the probes inserted into the crystallizer.

We selected the intense peak at 1601 cm^–1^, which corresponds to the aromatic vibration, ν_C=Cring_ of curcumin to quantify the solid concentration in the solution. This peak was selected in particular, as the intensity of this peak is more sensitive to the change in the solid-phase concentration. The ratio of the peak intensity with respect to the peak intensity of the solvent was found to be linearly proportional to each other and followed eq 1. The peak intensity of the curcumin here refers to the height of this band from the two-point baseline that connects 1617 and 1571 cm^–1^ in the Raman spectra.

### 5.5 Characterization of the Solid Phase

The morphological features of the curcumin particles were analyzed using SEM, high-resolution TEM, and optical microscopy. For SEM, the solid samples were transferred onto the carbon tape mounted on an SEM stage. Samples were coated with gold for 1 min, and the images were obtained using an SU70 Hitachi FEG-SEM instrument. For TEM analysis, curcumin spherulites were slightly crushed between two glass microscope cover slips, and the particles were then dispersed in hexane using ultrasound. The hexane solution that contains well-dispersed particles of spherulites was then transferred to holey carbon TEM grids (200 mesh Cu; Ted Pella, Inc, USA, lot # 031117) using a plastic dropper, and hexane was allowed to evaporate naturally at room temperature in a fume hood. For determining the structural details of the intermediate phase in the supersaturated solution, prior to nucleation, we used a different protocol that relies on a flash evaporation technique using a TEM grid placed on a hot plate at 95 °C. The (supersaturated) solution sample was collected roughly an hour before nucleation and drop cast on the hot TEM grid. Data were collected on a Thermo Fisher Titan Themis transmission electron microscope at 300 keV using a Gatan OneView detector.

A simple light microscope (Olympus) was used to capture the images of the spherulites collected from the solution during the experiment. These samples were collected from the crystallizer roughly 5 min after the onset of nucleation using a 1 mL dropper. The samples were immediately transferred to a glass cuvette, and the images were captured immediately under room temperature conditions. Typically, images were captured using a light microscope within 1 min after sampling.

PXRD analysis of the spherulites and FI curcumin was recorded using a PANalytical Empyrean diffractometer with Cu K_α1_ radiation (λ = 1.5406 Å) at 40 kV and 35 mA over the 2θ range of 5–40°, using a step size of 0.1° and total collection time of 15 min. The crystallinity of the spherulites and form I curcumin was confirmed by the flat baseline in the entire range of 2θ = 5–40°.

SAXS measurements were carried out at ambient temperature using a Bruker Nanostar diffractometer with Cu K_α1_ radiation (λ = 0.15406 nm) connected to a position-sensitive HiStar detector. Silver behenate (AgC_22_H_43_O_2_) has been used as a standard SAXS calibrant for evaluating the scattering vector, *Q*, from the sample-to-detector distance (*Q* = 4π sin θ/λ, where λ and 2θ are the wavelength and the scattering angle, respectively). The *Q* range varied approximately from 0.0135 to 0.3555 Å^–1^. Before adding the samples into the cell, they were evacuated in a glass cell up to 70 °C under high-vacuum conditions (up to 10^–7^ mbar) using a turbo vacuum pump for 24 h. The chamber was evacuated before measurement, and the sample was measured for 1000 s. Finally, the raw data were corrected for the instrumental background and scattering of the empty cell.

A Q2000 differential scanning calorimeter from TA Instruments was used to perform the thermal analysis of the recrystallized samples. Experiments were carried out using hermetically sealed aluminum pans, which contain a definite amount of each sample (∼5 mg). The experiments were performed over a temperature range of 25–190 °C at a heating rate of 10 °C min^–1^ under a N_2_ atmosphere (40 mL min^–1^). The results were analyzed as heat flow (W g^–1^) *versus* temperature (°C) using TA Instruments Universal 2000 software (Universal V4.5A).

A Malvern Zetasizer ZSP Nano instrument equipped with a temperature controller was used to measure the size of the intermediate phases before nucleation in the impure solution at three different time intervals by DLS. In this technique, measurement of the time-autocorrelation function of the intensity of laser light scattered by a sample is used to calculate an average diffusion coefficient. This, in turn, yields an estimate of the solvodynamic diameter of the scattering species. The mean solvodynamic diameter was estimated using the cumulant method. Measurements were made at a controlled temperature of 5 °C using a forward scattering angle of θ = 12.8° of laser light (λ = 632.8 nm) with automatic cell positioning and an automatic attenuator. All the solutions were allowed to equilibrate for 120 s at the measurement temperature inside the DLS cell before commencing the measurements. For every sample, five measurements, each consisting of 15 scans, were made. Intensity distributions were obtained from the autocorrelation function using multiple narrow modes. Data analysis was carried out using Malvern Zetasizer software v. 7.11.
